# Lighting pathways to success in STEM: a virtual Laboratory Meeting Programme (LaMP) mutually benefits mentees and host laboratories

**DOI:** 10.1098/rspb.2024.0149

**Published:** 2024-05-29

**Authors:** Katie E. Lotterhos, Moisés A. Bernal, Megan Phifer-Rixey, Torrance Hanley

**Affiliations:** ^1^ Northeastern University Marine Science Center, Nahant, MA 01908, USA; ^2^ Department of Biological Sciences, Auburn University, Auburn, AL 36849, USA; ^3^ Smithsonian Tropical Research Institute, Panama 0843-03092, Panama; ^4^ Department of Biology, Drexel University, Philadelphia, PA 19104, USA; ^5^ Department of Biology, Sacred Heart University, Fairfield, CT 06825, USA

**Keywords:** diversity, inclusion, hidden curriculum, academic networking

## Abstract

Developing robust professional networks can help shape the trajectories of early career scientists. Yet, historical inequities in science, technology, engineering, and mathematics (STEM) fields make access to these networks highly variable across academic programmes, and senior academics often have little time for mentoring. Here, we illustrate the success of a virtual Laboratory Meeting Programme (LaMP). In this programme, we matched students (mentees) with a more experienced scientist (mentors) from a research group. The mentees then attended the mentors’ laboratory meetings during the academic year with two laboratory meetings specifically dedicated to the mentee’s professional development. Survey results indicate that mentees expanded their knowledge of the hidden curriculum as well as their professional network, while only requiring a few extra hours of their mentor’s time over eight months. In addition, host laboratories benefitted from mentees sharing new perspectives and knowledge in laboratory meetings. Diversity of the mentees was significantly higher than the mentors, suggesting that the programme increased the participation of traditionally under-represented groups. Finally, we found that providing a stipend was very important to many mentees. We conclude that virtual LaMPs can be an inclusive and cost-effective way to foster trainee development and increase diversity within STEM fields with little additional time commitment.

## 1. Introduction

Robust support networks, particularly for undergraduate and graduate students, can boost short- and long-term success in scientific disciplines and shape career trajectories. Yet access to these networks can be highly variable within and across academic institutions, often perpetuating historical inequities in STEM. In particular, first-generation college students and students from marginalized groups are disproportionately impacted by limited interactions with peers and mentors, such as graduate students, postdoctoral scientists and/or faculty [1–4]. Students at community colleges and primarily undergraduate institutions (PUIs) may also have fewer networking opportunities than institutions with a higher proportion of graduate students, postdoctoral researchers and research faculty.

Knowledge of the ‘hidden curriculum’—unwritten standards across academic hierarchies—often depends on access to such professional networks [5,6]. The hidden curriculum encompasses a suite of skills beyond those traditionally acquired in a classroom setting that are critical to success in academia and beyond, including knowing how to apply to positions at all levels; learning how to write personal, research, and teaching statements; finding grant and fellowship opportunities; and refining applications and proposals. Lack of access to a professional support network during college may decrease the likelihood of undergraduate students applying to and being selected by graduate programmes [7,8]. Similarly, graduate students at PUIs or in smaller laboratories and departments may have fewer opportunities to develop, refine and diversify their professional skills as a result of having more limited networks. At all levels, the paucity of mentoring networks can also lead to questioning academic belonging and ultimately affect participation and engagement in the sciences [5]. Thus, finding novel ways to address inequities in access to academic support networks is essential to improve retention, and ultimately achieve long-term goals of increasing diversity, equity and inclusion in STEM fields.

Virtual laboratory meetings, mentoring programmes and research experiences can connect students and faculty across institutional boundaries, creating opportunities for the development of mutually beneficial relationships and professional networks [9–11]. The recent expansion of resources for virtual communication has the potential to decrease the insularity of academic institutions, broaden academic networks and create new opportunities for faculty and students. For example, during the COVID-19 pandemic, many seminar series were made available to interested students and faculties outside the host institutions [12,13]. In addition, workshops and training opportunities on topics ranging from genomics, to improving field safety, to demystifying graduate school switched from in-person venues to online communities that were broadly advertised and easily accessible. Virtual and hybrid scientific meetings have also become much more common, revealing both advantages and limitations of these virtual formats [14–18].

**Figure 1.**
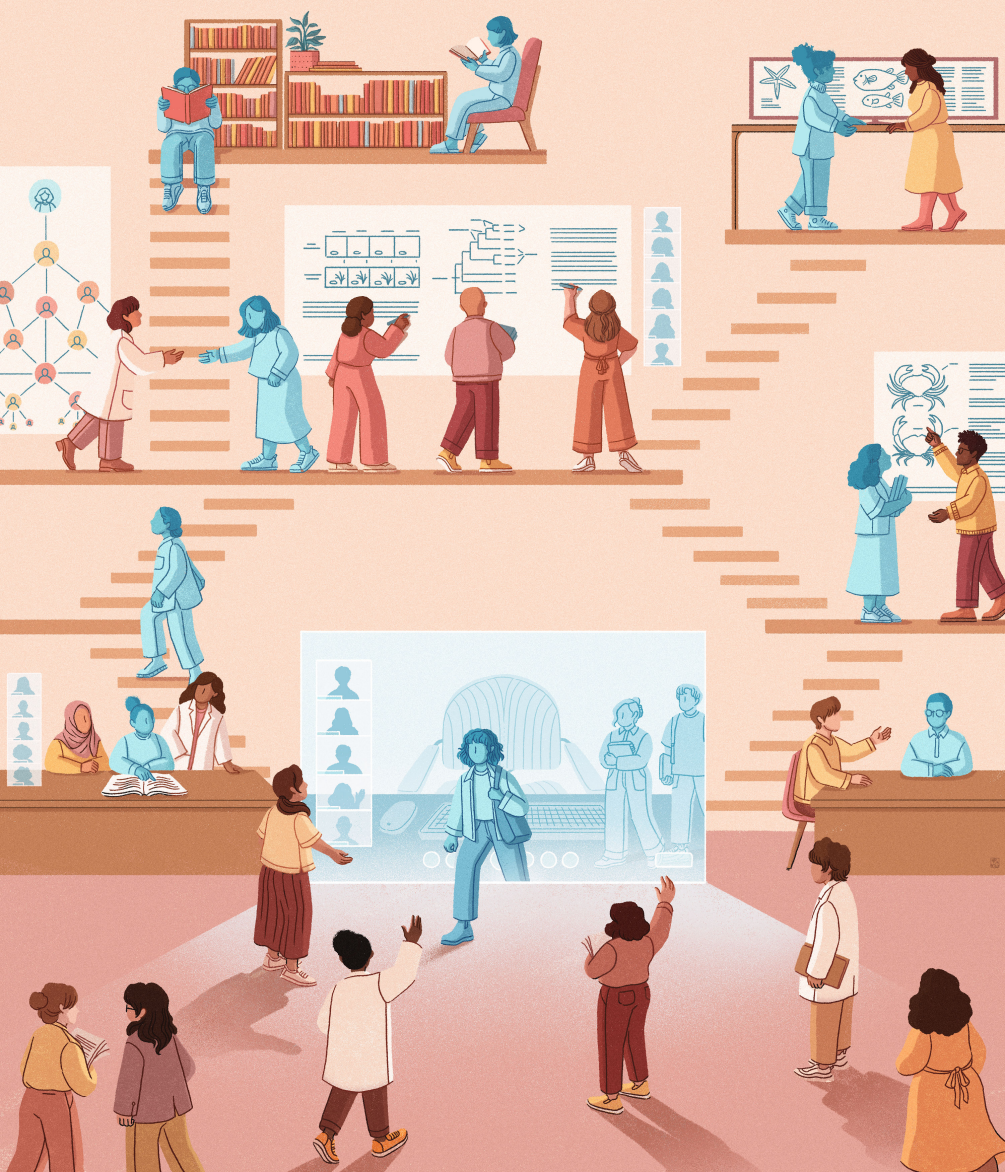
The virtual LaMP facilitates training and networking of the mentee (blue) through virtual interactions with the host research laboratory (warm colours). Illustration by Christina Chung.

The present study describes the virtual Laboratory Meeting Programme (virtual ‘LaMP’) developed by the Research Coordination Network for Evolution in Changing Seas (RCN-ECS). In this programme, the RCN sought to provide training to undergraduate and graduate students (from here on referred to as ‘mentees’) by giving them the opportunity to participate in laboratory meetings of a research group with similar research interests (figure 1). The programme aimed to help mentees expand their professional network, gain access to relevant information about diverse topics in academia, broaden their knowledge in the fields of evolution, ecology and marine biology, and build a foundation for future interactions and collaborations. Each mentee was paired with an established laboratory group and mentor (postdoctoral associate, research scientist or tenure-track faculty) conducting marine and/or evolutionary research. To successfully complete the programme, mentees had to attend at least 10 of the mentor’s laboratory meetings, two of which had to be dedicated to mentee professional development. The format for professional development meetings was flexible and depended on the needs of the mentee (e.g. graduate school applications, grant writing, research presentations, paper reviews or discussions on diversity, equity and inclusion in academia). At the end of the programme, eligible mentees received a US $500 stipend. The programme ran in the academic years of 2020–2021 (during the COVID-19 pandemic when laboratory meetings were remote), 2021–2022 (after lockdowns ended and meetings moved back to in-person), and 2023-2024.

Below, we report the results of a survey of mentees and mentors collected in 2022. We assess the success of the programme, share key takeaways, provide resources for running the programme and suggest potential modifications/improvements to inform future initiatives with similar goals. The results show that virtual LaMPs are a simple, cost-effective and time-effective model that could easily be adopted by any professional field to strengthen professional networks, increase diversity and facilitate translation of the ‘hidden curriculum’.

## 2. Methods

### (a) Running the virtual Laboratory Meeting Programme

**Figure 2.**
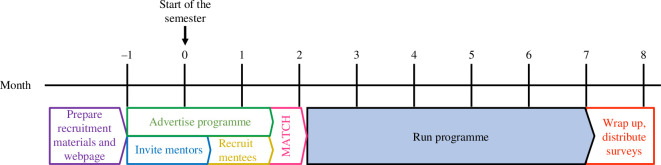
Approximate timeline for the Laboratory Meeting Programme. Data were collected in 2022 for cohorts 2020–2021 and 2021–2022.

The first three months of the programme require dedicated time for recruiting and matching mentees and mentors (for summary of programme timelines see figure 2). One month prior to the start of the academic year, we began to advertise the programme by sharing a link to our webpage with potential mentors and mentees (https://rcn-ecs.github.io/VLMTP/; figure 2). Then, we recruited mentors through a list of personal invitations, listservs and members of the RCN-ECS. We completed the process of recruiting 30–40 mentors approximately two weeks after the academic year started (figure 2). Mentors were required to agree with a document that outlined expectations and best practises for including their mentee in laboratory meetings (see the electronic supplementary material).

After mentors were recruited, we began the process of recruiting student mentees. We generated an email contact list with the goal of contacting as many participants as possible across a diverse group of scientific societies and institutions. The contact list consisted of scientific societies or diversity lists (e.g. Society for Advancement of Chicanos/Hispanics & Native Americans in Science, Diversify Ecology and Evolutionary Biology, Black Women in Ecology Evolution and Marine Science, American Geophysical Union BRIDGE, Association for the Sciences of Limnology and Oceanography Multicultural programme, Ecology Society of America SEEDs programme, Asian Americans & Pacific Islanders in Geosciences) and a list of 608 professors teaching courses in biology, ecology or evolution at Historically Black Colleges and Universities, Hispanic Serving Institutions, and Tribal Colleges and Universities. In addition, we advertised to the RCN-ECS listserv and asked colleagues to distribute the information among peers.

To apply, mentees submitted a 300-word statement that described: (i) their current research interests and/or experiences related to the themes under the RCN-ECS; (ii) future career interests; (iii) how interactions with a host laboratory would help to advance their careers and/or support their professional development; and (iv) a description of how their participation in this programme would help to increase diversity (broadly defined) within the network. They also: (i) answered questions about their time zone; (ii) listed their top three choices for mentors; (iii) selected two keywords that described their research interests from a list; (iv) submitted a CV or resume; and (v) optionally answered demographic questions.

Approximately six weeks into the academic year, we closed applications for mentees and started pairing them with mentors. Matching was made by two members of the RCN diversity committee based on the mentee’s academic interests, who they listed as their top three choices for a mentor, and time zone alignment, taking into account how many mentees could be assigned to a single mentor (i.e. usually 1–2 mentees per laboratory group). Owing to high request rates for well-known mentors, sometimes we were unable to match a mentee with one of their top three choices. In the few cases, where mentees did not get their top choices, pairings were made based on affinity between mentors’ and mentees’ research interests. By the second month of the academic year, we had completed the process of pairing mentees with mentors. Pairs were introduced to each other by email and reminded of the programme guidelines and expectations (see the electronic supplementary material for example emails).

Over the course of the academic year, mentees attended laboratory meetings on an independent basis. At the end of the academic year, we distributed stipends to students for their participation in the programme. To obtain a stipend, students had to provide a letter from their mentor that stated the student had completed the programme requirements.

### (b) Mentee and mentor surveys

At the end of the academic year in 2022, we distributed surveys to mentees and another survey to mentors, who had participated in the programme (for complete surveys, see the electronic supplementary material). Both surveys included optional questions on demographic information, year(s) of participation, activities that were part of laboratory meetings, potential for future collaborations, a Likert scale on how they ranked the programme from 1 to 10 and open-ended feedback (table 1, left column). We also had an open-ended question where participants were encouraged to leave constructive feedback.

**Table 1.** Shared and unique questions for the mentee and mentor surveys are listed in columns.

both mentees and mentors	unique to mentee survey	unique to mentor survey
career stage	degree of agreement with statements regarding professional network and knowledge	number of mentees trained to date
gender identity	importance of stipend to completing the programme	professional development activities discussed with mentees
racial identity	what kind of interactions advanced professional development	contribution of mentees to laboratory meeting
sexual orientation	what kind of knowledge was gained	time investment in programme
disability	continued interactions with host laboratory	career stage of mentee
year of participation in the programme		attendance of mentees beyond programme requirement
activities included in laboratory meeting		number of people at laboratory meetings
future collaboration		1 : 1 interactions with other laboratory members
rate the programme on a scale from 1 to 10		degree of agreement with statements regarding continued interactions and benefits of having the mentee
open-ended feedback		

The mentee survey included unique Likert scale questions on whether the programme helped them extend their professional network, whether they advanced their expertise in subject matter, and how important the stipend was to completing the programme. We also asked mentees what kind of interactions most helped to advance their professional development, what knowledge they gained during the programme, and whether they planned to continue interactions with the host laboratory (table 1, middle column).

The mentor survey included questions on the number of mentees hosted, professional development topics discussed in laboratory meetings, mentee contributions to laboratory meetings, the degree of agreement with statements regarding continued interactions and benefits of having the mentee join laboratory meetings, how much time mentors invested in the programme, whether mentees attended laboratory meetings beyond the programme requirements, and how many people attended their laboratory meetings (table 1, right column).

### (c) Institutional Review Board review

Our surveys were reviewed by the Institutional Review Board (IRB) at Northeastern University (IRB no.: 22-03-33) and were considered exempt (DHHS Review Category: EXEMPT, CATEGORY no. 2 Revised Common Rule 45CFR46.104(d)(2)(iii)).

### (d) Statistical analysis

To determine if the diversity of mentees was significantly different from mentors, we tested the null hypothesis of independence between mentor/mentee status and (i) gender, (ii) race, or (iii) sexual orientation with Fisher’s exact tests. Other survey questions were summarized by the number or percentage of respondents in each response.

## 3. Results

### (a) Participation in the programme and the survey

Data presented are from participants in the programme for the 2020–2021 (year 1) and 2021–2022 (year 2) academic years (eight months; figure 2) who responded to the survey at the end of the 2022 academic year (table 1). All the mentees who applied to the programme were matched to a mentor. In year 1, we had 33 mentors and 35 mentees; and in year 2, we had 28 mentors (many returning for a second year) and 39 mentees (all new to the programme). Only a few mentees could not be placed with one of their choices for mentors. While mentors almost exclusively worked at United States (US) institutions, 85% of mentees were US citizens and the remaining 15% were from South America, Asia, the Middle East or Europe. Mentees could have been attending a US or international institution, as this was not included in the survey. The mentor survey had 35 responses total (11 participated in both years; nine in year 1 only; and 15 in year 2 only). The mentee survey had 41 responses with the majority of respondents participating in year 2 (nine had participated in year 1 and 33 participated in year 2).

### (b) Diversity of participants

**Figure 3.**
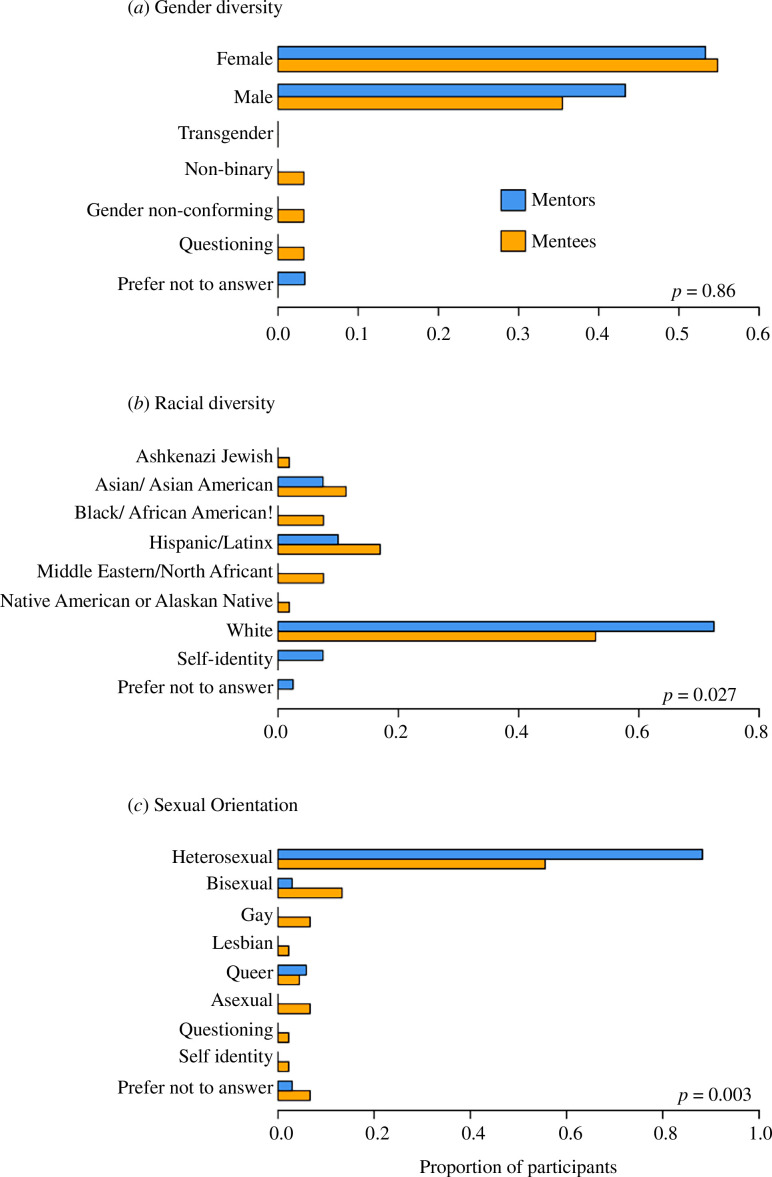
Gender representation was similar among mentees and mentors (*a*), but mentees were more diverse than mentors with respect to (*b*) racial identity and (*c*) sexual orientation. The proportion of mentees/mentors is based on survey responses from two cohorts (years 2020–2021 and 2021–2022).

Participants had the option in the survey to report their gender, race and sexual orientation with open options (figure 3). Most of the participants identified either as male or female (figure 3*a*), and there was no relationship between mentor/mentee status and reported gender (Fisher’s exact test, *p* = 0.86). However, we rejected the null hypothesis of independence between mentor/mentee status and racial diversity (Fisher’s exact test, *p* = 0.027), indicating that the proportional representation of different races in the mentors versus mentees was statistically significant (figure 3*b*). The proportion of respondents who identified as a member of a group under-represented in STEM was higher in mentees compared with mentors, with a lower proportion of mentees selecting ‘White’ compared with mentors (figure 3*b*). There was also a statistical difference in the representation of different sexual orientations in the mentors versus mentees (Fisher’s exact test, *p* = 0.003; owing to low sample sizes in some of the survey categories, participants were grouped into two groups: (i) heterosexual and (ii) lesbian, gay, bisexual, queer, asexual, questioning, and self-identity, figure 3*c*). The proportion of heterosexual participants was higher in mentors compared with mentees, with several mentees identifying as members of the LGBTQ+ community (figure 3*c*).

### (c) Programme outcomes

**Figure 4.**
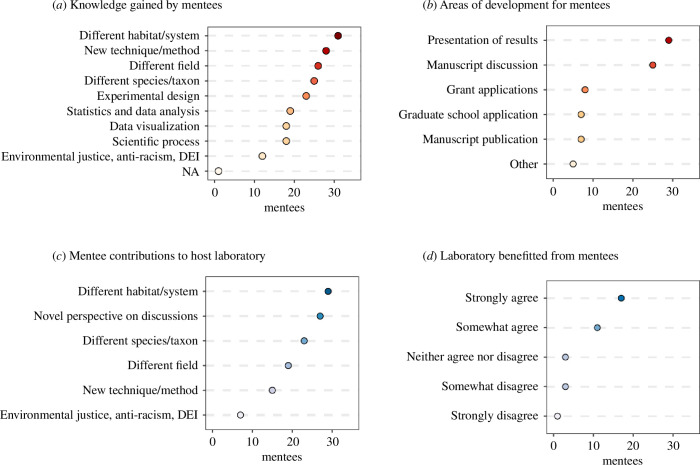
Assessment of how mentees (red dots) and mentors/laboratories (blue dots) benefitted from the programme, with the darkness of the shade corresponding to the number of responses. The number of mentees/mentors is based on survey responses from two cohorts (years 2020–2021 and 2021–2022). Mentee survey responses are shown for (*a*) areas of knowledge development, and (*b*) areas of professional development addressed in laboratory meetings (mentees selected all that applied for each question). Mentor survey responses are shown for (*c*) areas that mentees contributed to laboratory meetings (mentees selected all that applied), and (*d*) level of agreement with the statement ‘members of my laboratory benefitted from participating in this programme’. DEI, diversity, equity, and inclusion.

According to the surveys, some of the most important outcomes of the virtual programme were: (i) the knowledge gained by the mentees; (ii) the professional development gained by the mentee; and (iii) the contributions of the mentee to the laboratory meeting. We asked mentees what kind of knowledge they gained by attending laboratory meetings and gave them a list of options with instructions to check all that apply (figure 4*a*). More than half of the mentees gained knowledge of a different field, habitat, system, species, technique or method, as well as the knowledge of experimental design (figure 4*a*). In addition, almost half of Mentees (44%) reported gaining knowledge in statistical analysis, data visualization or the scientific process.

To assess gains in professional development, we asked mentees: ‘in what areas did the interactions with the host laboratory help advance their professional development?’ The majority of mentees said that laboratory meetings advanced their understanding of how to present research (71%) and how to discuss a published paper (61%; figure 4*b*). A smaller percentage of mentees (15–20%) said that laboratory meetings also helped them learn how to apply for grants, how to apply/interview for graduate school and/or how to publish a paper (figure 4*b*). Some mentees also ticked ‘Other’ and responded that laboratory meetings helped them learn how to network in the field, how to start learning statistics, how to get a postdoctoral appointment, and how to get feedback on presentations.

We asked mentors about mentees’ contributions to laboratory meetings, with instructions to tick all the options that applied. The majority of mentors (77%) said that mentees brought a novel perspective on discussions to their laboratory (figure 4*c*), and more than half of mentors responded that mentees brought new knowledge of a habitat, system, species or field (figure 4*c*). For both mentor and mentee surveys, anti-racism and diversity, equity, and inclusion (DEI) were the least discussed topics (figure 4*a,c*). Overall, mentors agreed that their laboratory benefitted from having a mentee through the duration of the programme (figure 4*d*).

### (d) Developing professional networks

**Figure 5.**
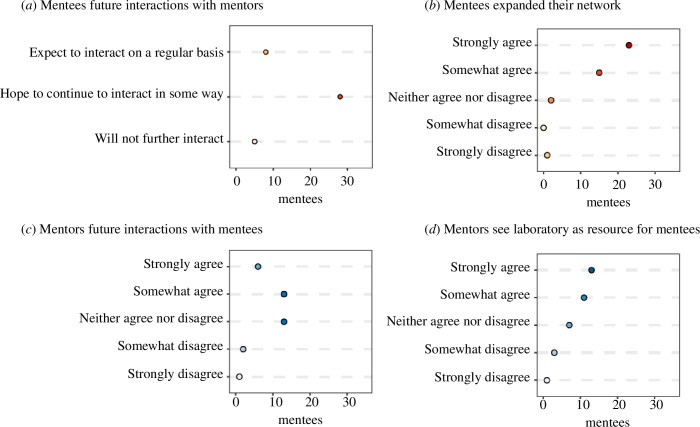
Expectations of future interactions or collaborations, from the perspective of mentees (red dots) and mentors (blue dots), with the darkness of the shade corresponding to the number of responses. The number of mentees/mentors is based on survey responses from two cohorts (years 2020–2021 and 2021–2022). Mentee survey responses are shown for (*a*) future interactions with mentors, and (*b*) level of agreement with the statement ‘the programme helped me to extend my professional network’. Mentor survey responses are shown for the level of agreement with the statements (*c*) ‘I plan to interact with the mentee in the next year’, and (*d*) ‘my laboratory will be a resource to the mentee after the completion of this programme’.

Regular meetings with mentors and laboratory members have the potential to develop and strengthen professional networks. We asked mentees if they expected to have future interactions or collaborations with mentors, and asked mentors whether they will continue to interact with and be a resource to mentees. Approximately 68% of mentees hoped to interact in some way in the future, 20% expected to continue to interact with their host laboratory on a regular basis and 12% said they would not interact in the future (figure 5*a*). The vast majority (93%) of mentees agreed that the programme helped them expand their professional network (figure 5*b*). Expectation of future collaboration was more limited, with approximately 12% of mentees responding that they definitely expected to collaborate with mentors and their research groups and 22% indicating probable collaborations. While most mentors did not indicate strong agreement with the statement ‘I plan to interact with the mentee in the next year’ (figure 5*c*), very few disagreed with the statement and 77% of mentors agreed that their laboratory would be a resource for mentees in the future (figure 5*d*).

### (e) Resource commitment

**Figure 6.**
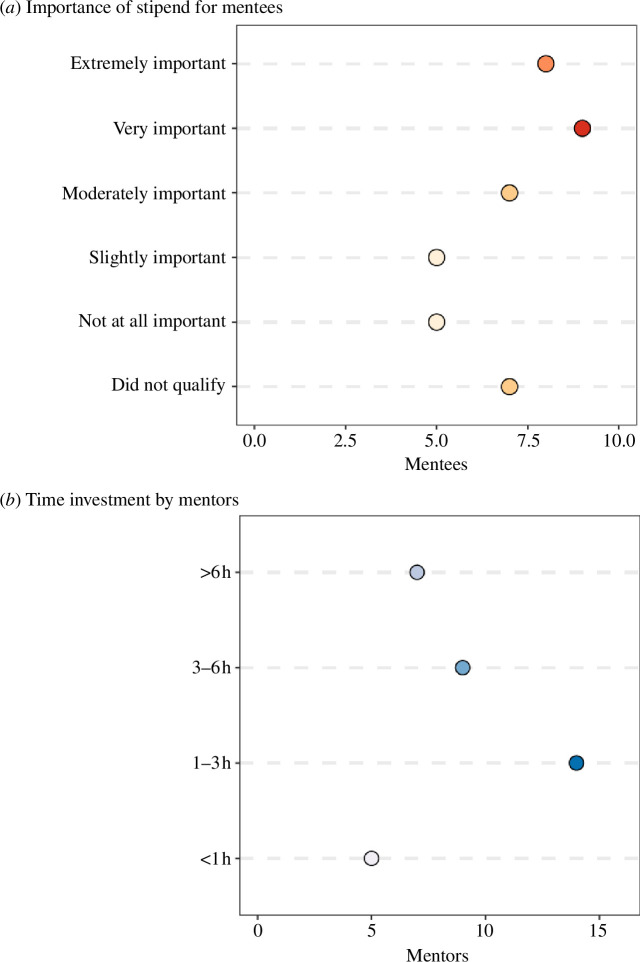
Resources committed to the programme. The number of mentees/mentors is based on survey responses from two cohorts (years 2020–2021 and 2021–2022). (*a*) Mentee responses (red dots) to the question: ‘how important was the stipend to your completion in the programme?’ (*b*) Mentor responses (blue dots) to the question: ‘without counting the amount of time you normally spend on laboratory meetings, how much time did you invest towards your participation in this programme in each year?’. The darkness of the shade corresponds to the number of responses.

The major monetary commitment for the programme was the stipend for mentees that completed the minimum number of virtual meetings ($500). We asked the mentees how important the stipend was towards the completion of the programme, and 59% of respondents said it was moderately, very or extremely important (figure 6*a*). An additional 24% said it was slightly or not important, and the remaining respondents did not qualify for a stipend owing to their citizenship (figure 6*a*).

The major non-monetary resource commitment was the amount of time invested by mentors. The majority of mentors spent 1–3 h on the programme over the course of an academic year, in addition to the time they normally spent on laboratory meetings (figure 6*b*). Meanwhile, in terms of programme administration, the majority of the resource commitment was volunteer’s time. Around 20–40 person hours were required for programme set-up, which included advertising the programme to potential mentors, processing mentor applications, setting up the website including a public list of mentors and application materials and advertising to potential mentees. Another 5–10 person hours were required to match mentees with mentors, and to communicate to both parties expectations of the programme. After the matching was completed, there was little programme administration outside of occasional check-ins. At the conclusion of the programme, another 20–40 person hours was required for processing stipends. Templates for running the programme (e.g. committee responsibilities, email templates and forms) can be found on the *Evolution in Changing Seas RCN* website at https://rcn-ecs.github.io/VLMTP_Supp/, and can help reduce the time commitment in setting up a programme for the first time.

### (f) Overall programme assessment

**Figure 7.**
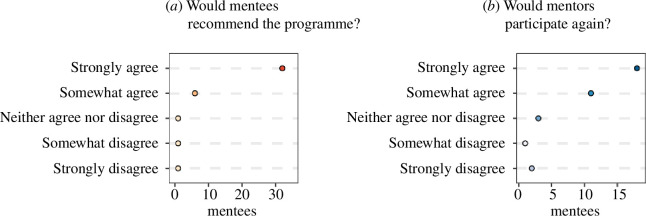
Assessment of the programme by mentees (red dots) and mentors (blue dots), with the darkness of the shade corresponding to the number of responses. The number of mentees/mentors is based on survey responses from two cohorts (years 2020–2021 and 2021–2022). (*a*) Mentee’s level of agreement with the statement ‘I would recommend this programme to a friend’. (*b*) Mentor’s level of agreement with the statement ‘I would participate as a mentor in this programme again’.

To get an overall opinion of the programme, we asked mentees whether they would recommend this programme to a friend and we asked mentors whether they would participate in the programme again. There was a strong level of agreement for both parties on those questions (figure 7). We also asked mentors and mentees to rate the programme on a scale of 1 (lowest rating) to 10 (highest rating). The average rating for mentors was 8.1, while the average rating for mentees was 8.5. We asked mentors if their mentees attended more meetings beyond the requirement for a stipend (10 meetings), and 80% responded that their mentee attended or planned to attend sessions beyond the requirement.

In the open-ended question asking for feedback, many mentees were enthusiastic about the programme and articulated how the programme advanced their professional network, advanced their career through feedback and new collaborations, and gave them new insight into a different field (table 2). Some of the constructive feedback received included: difficulties with scheduling, mismatches between the interests of mentors and mentees, and a lack of diversity in topics covered in laboratory meetings (table 2).

**Table 2.** Mentee and mentor responses to the open-ended survey question. (Some responses have been paraphrased to protect the anonymity of the respondent.)

topic	positive feedback	constructive feedback
network development	(mentee) ’it was nice to be able to participate in a lab meeting that had research interests that overlapped with mine as this opportunity was not available … at my current institution’. (mentee) ‘I think having this program in the middle of the pandemic helped a little bit to put some sense of community among marine ecologists that we, students, felt lacking’. (mentor) ‘we hosted two EXCELLENT mentees this year. They greatly enriched our discussion, and I think they also benefitted. Both chose to participate in additional meetings after completing their formal requirements’.	(mentee) ‘my mentor did not regularly schedule meetings … [I] was not paired with any of the people that I had ranked, nor did my mentor’s interests align with mine’. (mentor) ‘it would have been useful to the mentees and mentor labs to have some guidance on expectations and some ongoing assessment (checkpoints) through the program’.
career advancement	(mentee) ’the program propelled my career in a new direction. I am currently collaborating with my host lab through [a working group]. It has led to [a publication] and a new aspect of my research’. (mentee) ‘I was able to practice giving a presentation that I would later deliver to [a potential advisor]’. (mentee) ‘helped a lot with understanding grant funding and what I will likely encounter in the future’.	(mentor) ‘I don't really know why they chose to participate in this program but they did not need anything, they were not interested in our research, had already quite clear plans on what to do after they graduate, did not need any help with their research [or applications] …’. (mentor) ‘[the mentee] wasn't clear on what was happening, what they wanted to get out of it, or how we could support them, and I don't think it was a particularly useful experience for them’.
laboratory meeting content	(mentee) ‘I really got a lot out of this program. It was exciting to learn about research going on in a similar field with different approaches and study organisms. Also, really interesting to see another PI’s mentoring style’. (mentee) ‘gained insight into a field which I would otherwise not have the opportunity to interact with. Learned how to present on peer-reviewed papers and lead discussions’.	(mentee) ‘I would have liked a bit more diversity in the types of lab meetings, since most of the meetings were about discussing papers or grant proposals’.
scheduling		(mentee) ‘While this program didn't hurt, I also don't think it helped much. [It was] more difficult than I thought it would be to balance attending both my lab meetings and the RCN ECS host lab meetings weekly’. (mentor) ‘it was difficult for the student to attend all lab meetings due to a scheduling conflict with his classes’. (Mentor) ‘scheduling lab meetings each semester can be a challenge, and one thing that happened is with mentees in different time zones and with one of the mentees an undergrad with a heavy class schedule, finding a time for the spring meeting time was VERY difficult’.

## 4. Discussion

### (a) Success of the virtual Laboratory Meeting Programme initiative

Overall, data strongly support that LaMP achieved its primary goals. Survey results demonstrate that participating in cross-institutional, virtual/hybrid laboratory meetings can provide mentees with access to professional support networks, increase their knowledge of the hidden curriculum and provide field-specific training. Specifically, the vast majority of mentees agreed that the programme helped them expand their professional network, noting that it created a ‘sense of community’ and offered an ‘opportunity that was not available … at [their] current institution’. Lack of access to a robust support network disproportionately impacts first-generation college students [3] and students from systematically marginalized groups [1,2,4], often decreasing their feelings of academic belonging and comfort with professional networking [5]. LaMP helped redress this inequity by creating opportunities for students to communicate directly with scientists at multiple career stages in their field(s) of interest, reducing both real and perceived academic barriers. Reducing barriers is particularly important for students at colleges with fewer research opportunities and/or smaller programmes (e.g. schools lacking a marine biology major or molecular facilities).

The programme was also mutually beneficial for mentees, mentors and the laboratory groups. For example, one mentee noted that ‘the program propelled [their] career in a new direction’ and one mentor noted that their ‘two EXCELLENT mentees … greatly enriched [the lab’s] discussion’. A key takeaway from the mentee survey was that the programme provided a unique opportunity for them to learn about different fields of study, current methods and techniques, and how an academic laboratory is run. A key takeaway from the mentor survey was that their laboratory benefitted from the mentee’s knowledge of a different system or species, as well as the mentee’s novel perspective on discussions. This exchange of knowledge has the potential to increase access to different research fields and perspectives for all participants. This highlights how the programme can be beneficial not only for mentees but also for mentors and their research groups.

In addition to supporting mentees, one of the main objectives of this programme was to increase the participation of marginalized groups in the sciences. Both marine science and evolutionary biology are notable for their lack of diversity [19,20], and related fields like geosciences have observed little increase in diversity in the last 40 years [21]. LaMP did successfully engage mentees with diverse identities and backgrounds. Mentees were more diverse than mentors in terms of racial identity and sexual orientation, reflecting the general disparity between students and faculty in STEM fields [22,23]. However, there is still progress to be made, and better integrating PUIs, community colleges, liberal arts colleges and international universities in virtual meeting programmes could further broaden participation (see §4.2).

Results from the survey indicate that the recurring nature of laboratory meetings over the academic year laid the groundwork for robust and lasting network connections, with most mentees interested in future interactions and collaborations with mentors and/or laboratories, and most mentors willing to serve as a resource in the future. Mentees also gained knowledge of the hidden curriculum through this programme, getting feedback on research presentations, grant proposals, graduate school applications and manuscript drafts. This initiative adds to a growing list of programmes that explicitly address the hidden curriculum and help students develop diverse skills in STEM, including first-year seminars focused on the ‘epi-curriculum’ [2]; online, cross-institutional journal clubs [2]; and research experiences like the National Summer Undergraduate Research Project [9,11]. Such opportunities are particularly important for first-generation students who may have fewer interactions with faculty owing to lack of knowledge of implicit cultural norms and/or perceived barriers [24].

Previous studies have highlighted how virtual formats can increase access to professional networks, despite some of the downsides of virtual formats [16–18]. The downsides of virtual formats include technical requirements and ‘zoom fatigue’ that occurs during prolonged online engagement such as virtual conferences [14,15,25–27]. On the other hand, virtual programmes such as this LaMP avoid zoom fatigue by offering short, but intensive and ongoing interactions. Our results and those from the National Summer Undergraduate Research Project, which connects Black, Indigenous, People of Colour (BIPOC) and Hispanic undergraduates in STEM fields through remote summer experiences [9,28], add to a growing body of evidence that repeated short virtual interactions with small research groups are an effective way to engage dozens of participants at low cost.

### (b) Considerations for future programmes

While the virtual LaMP was successful in many ways, we also identified room for improvement in the areas of diversity, logistics and ensuring and assessing long-term success that can be ameliorated by following simple rules for mentoring programmes [29]. LaMP was successful in recruiting a mentee pool that was more diverse than the mentor pool, but increasing the diversity of both types of participants will continue to be important for the success of the programme. In particular, students from systematically marginalized groups benefit from developing relationships with mentors who share aspects of their identity [2,30]. While we did work to include diverse mentors and mentees by advertising via email, we believe the diversity of the participant pool could be extended even further by building long-term relationships with faculty and administrators from Historically Black Colleges and Universities, Hispanic Serving Institutions, Tribal Colleges and Universities, PUIs and community colleges [2,31].

There were also specific areas that could be improved for future iterations of the programme. Some of the more salient issues discussed by mentees and mentors were: scheduling conflicts, mismatched interests, and hybrid formats. Scheduling issues could be ameliorated in the future by asking mentors to provide a list of potential laboratory meeting times when they sign up for the programme, and requiring mentees to choose from mentors whose laboratory meeting time matches the indicated availability. Although mentees and mentors were matched based on their interests, mentees could not always be placed with their top choice and sometimes this worked out well (mentee learned a new study system or technique relevant to their career goals) and sometimes not (mentee was not engaged with the laboratory). In the future, we recommend evaluating mentee–mentor matches as an ongoing process that includes regular engagement with programme leadership, where mentees or mentors can provide feedback or voice concerns [29]. Finally, we found that hybrid meetings were most effective when all participants, remote or in-person, logged into the online meeting and shared their video. Doing so ensures that each person’s face can be seen by the remote participants on the call, helps remote participants feel like they are part of the group and facilitates captioning for those with auditory disabilities.

Perhaps the largest challenge for future virtual programmes will be securing the necessary funds associated with the meetings. The vast majority of participants that responded to the survey felt the stipend was very important for their participation and completion of the programme. Moreover, funding for international participants can be particularly challenging, because some federal grants only allow payment to citizens. With this in mind, this study highlights the need for professional societies and private foundations to expand funding for virtual meeting programmes that specifically provide funding for international participants. We recommend that funding also incentivizes future in-person interactions beyond the extent of virtual laboratory meetings, such as site visits to the mentor’s institution, workshops or conferences.

Finally, evaluation via an IRB survey is essential to sharing results and can broaden the impacts of the programme. Our survey focussed on assessing the short-term impacts of the programme. For future programmes, we suggest additional questions focused on long-term impacts, such as whether the programme increased feelings of belonging in the field for mentees [32] or whether they planned to remain in academia in the future.

## 5. Conclusions

Overall, our data suggest that virtual LaMPs like ours have significant benefits for early career scientists, as well as mentors and their laboratory groups. These benefits come at relatively low cost, with a small time investment from mentors and a modest monetary investment for mentee stipends. As organizations develop programming to recruit and retain diverse talent in STEM fields, virtual mentoring programmes are a cost-effective and time-effective model that can be adopted by any professional field to strengthen professional networks, increase diversity and facilitate translation of the ‘hidden curriculum’.

## Data Availability

Information and templates needed to run the programme are publicly available at [33]. To protect the anonymity of survey participants, survey data were summarized in each category and these summaries are archived on Dryad [34]. Electronic supplementary material, including the mentee and mentor survey, is available online [35].
